# SPTA1-Related Hereditary Spherocytosis: Novel Compound Heterozygous Mutations With Severe Clinical Manifestation

**DOI:** 10.7759/cureus.83724

**Published:** 2025-05-08

**Authors:** John Khor, Yang Liang Boo

**Affiliations:** 1 Internal Medicine, Hospital Sultanah Nora Ismail, Batu Pahat, MYS; 2 Hematology, Hospital Sultanah Aminah, Johor Bahru, MYS

**Keywords:** hemolytic anemia, hereditary spherocytosis, novel mutation, spta1 gene, transfusion

## Abstract

Hereditary spherocytosis (HS) is a common hereditary hemolytic anemia in which red blood cells (RBCs) assume spherocytic morphology, predisposing to easy destruction in the spleen. Diagnosis is readily made when spherocytes are demonstrated in the blood film of patients presenting with anemia, jaundice, and splenomegaly. A positive osmotic fragility test (OFT) is also supportive. However, when classical features are not seen in either blood film or OFT, diagnosis might become complicated, particularly in resource-limited settings. We report a teenager who was transitioned to the adult medical outpatient department with a diagnosis of hemolytic anemia; the etiology, however, was never identified. He has required monthly transfusions since six months of life and has developed antibodies to RBCs, secondary hemochromatosis, and gallstones. Workup for thalassemia and autoimmune causes was negative. A barrage of negative investigations ultimately led to a genetic analysis, which revealed a heterozygous mutation for the c.2671C>T (p.Arg891*) variant in the SPTA1 gene and a novel mutation for SPTA1 c.7134+5G>A (intronic). He was formally diagnosed with HS and underwent splenectomy. Post procedure, his anemia improved, and transfusion requirements steadily reduced. His sister exhibits the same heterozygous mutation for SPTA1 c.7134+5G>A (intronic), but did not have any clinical or laboratory manifestation of the disease. We postulate that this novel mutation in SPTA1 c.7134+5G>A (intronic) might play a role in determining disease severity, particularly when associated with a pathogenic variant.

## Introduction

Red blood cells (RBCs) are typically biconcave in shape, held together by five membrane proteins: band 3, protein 4.2, ankyrin, and α and β spectrin proteins [1]. In hereditary spherocytosis (HS), spherocytes are formed due to loss of membrane as a result of quantitative defects in these proteins that link the cytoskeleton to lipid bilayer (“vertical linkage”) [2]. These RBCs are generally sequestered and destroyed in the spleen, manifesting clinically as anemia, jaundice, and splenomegaly. Splenectomy forms the basis of treatment in patients with severe HS to ameliorate the symptoms and transfusion requirements [3].

HS is an autosomal disease, with autosomal dominant (75%) mutations occurring in either ankyrin (ANK1), band 3 (SLC4A1), or β-spectrin (SPTB) genes [3]. Mutations of SPTA1 are a rare cause of HS, comprising only 5% of affected HS cases, and are recessive in the majority of cases [3]. Most mutations are sporadic, unique, and without a familial pattern. Here, we report a case of a young adolescent with childhood hemolytic anemia of diagnostic challenge.

## Case presentation

A 19-year-old boy was diagnosed with hemolytic anemia at six months of life. Since then, he has been regularly transfused and investigated extensively for potential causes of hemolytic anemia. His peripheral blood film revealed occasional spherocytes. Alpha and beta globin chain deoxyribonucleic acid (DNA) analysis was negative for significant mutation. Glucose-6-phosphate dehydrogenase enzyme activity and osmotic fragility test (OFT) were normal. Bone marrow aspiration and trephine biopsy did not reveal any significant abnormality except erythroid hyperplasia. Family history did not reveal any relatives with hemolytic anemia. Given a chronic transfusion history, he developed alloimmunization and secondary hemochromatosis.

Upon review, his stature was short, with easily appreciated pallor and jaundice. Significant splenomegaly (20 cm from the left costal margin) but no hepatomegaly was found in the abdomen.

A repeat workup was done, which included a full blood count (Table 1) and basic biochemistry (Table 2), noting prominent anemia, significant indirect hyperbilirubinemia, and raised lactate dehydrogenase and ferritin. Magnetic resonance imaging T2* revealed moderate liver iron loading. Paroxysmal nocturnal hemoglobinuria screening and Coombs test were both negative.

**Table 1:** Full blood count. MCV: mean corpuscular volume; MCH: mean corpuscular hemoglobin; MCHC: mean corpuscular hemoglobin concentration.

Parameters	Value	Normal range	Unit
Hemoglobin	6	13.5 – 17.0	g/dL
White blood cells	4.61	4000 – 11000	/μL
Hematocrit	28.5	40.0 – 51.0	%
MCV	81.4	83.0 – 101.0	fL
MCH	27.7	27.0 – 32.0	pg
MCHC	34.0	31.5 – 34.5	g/dL
Red blood cells	3.5	3.80 – 4.80	×10^6^/μL
Platelets count	91	150 – 400	×10^3^/μL
Mean platelet volume	8.9	6.8 – 9.4	fL

**Table 2:** Basic biochemistry. ALT: alanine transaminase; ALP: alkaline phosphatase; LDH: lactate dehydrogenase; eGFR: estimated glomerular filtration rate.

Parameters	Value	Normal range	Unit
Total protein	67	67 – 83	mg/dL
Albumin	52.2	35 – 55	mg/dL
Total bilirubin	147	5 – 21	umoL
Indirect bilirubin	100	5 – 21	umoL
ALT	25.8	7 – 56	U/L
ALP	150	40 – 150	U/L
LDH	354	135 – 225	U/L
Blood urea nitrogen	3.0	5 – 8	mmol/dL
Sodium	141	135 – 145	mEq/L
Potassium	4.7	3.6 – 5.5	mEq/L
Chloride	100	95 – 105	mEq/L
Creatinine	54	78 – 118	mg/dL
eGFR	95	>60	mL/min/1.73 m^2^
Calcium	2.4	2.2 – 2.6	mmol/dL
Ferritin	903	24 to 336	ng/mL

He was eventually counseled for genetic study, and a subsequent report revealed a heterozygous mutation for c.2671C>T (p.Arg891*) variant in the SPTA1 gene. An intronic mutation (c.7134+5G>A) was also detected in his other SPTA1 allele. The same heterozygous mutation for SPTA1 c.7134+5G>A (intronic) was also found in his sister, but she did not have any clinical or laboratory manifestation of the disease.

**Figure 1:**
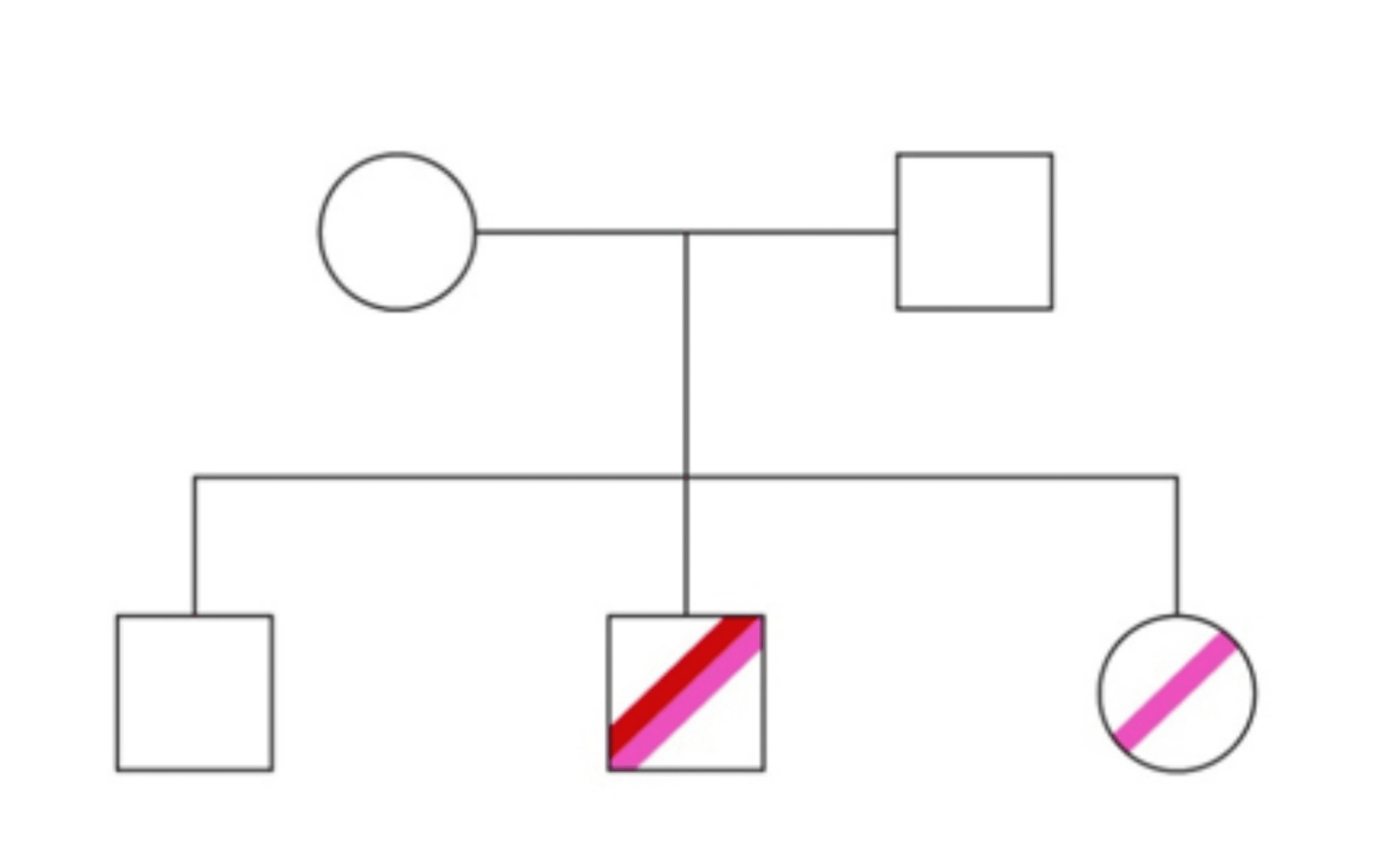
Family tree depicting the SPTA mutation in our patient’s family. The red line denotes c.2671C>T (p.Arg891*) variant, while the pink line denotes c.7134+5G>A (intronic).

The genetic analysis allowed a diagnosis of HS; as such, splenectomy with concurrent cholecystectomy was performed (the patient also had gallstone disease). Post splenectomy, his hemoglobin rose above 10 g/dL, and he was transfusion-free, with significant improvement in quality of life. A limited screening genetic test was done for his siblings and is illustrated in Figure 1.

## Discussion

SPTA1 is a gene located at 1q23.1 [4]. Two normal SPTA1 alleles encode for α spectrin, a protein responsible for the vertical integrity of RBC scaffolding. α spectrin interacts with β spectrin (responsible for horizontal integrity of RBC scaffolding) to maintain normal RBC morphology and is produced in excess of that of β spectrin [5]. In current literature, two common pathological alleles are reported in the SPTA1 gene mutation, i.e., α LEPRA and α LELY, and neither is detected in our patient. The presence of such alleles is known to reduce, but not eliminate α-spectrin expression [6,7]. Furthermore, given the excess production of α spectrin by a normal SPTA1 allele, the presence of heterozygous α LEPRA or α LELY allele, coupled with a normal SPTA allele, usually does not have significant clinical manifestation [6,7]. A significant reduction of α-spectrin production to less than 8% of normal levels usually leads to an increase in transfusion requirement in clinically significant HS [8,9].

The presence of homozygous mutation or compound heterozygous mutations, frequently involving the α LEPRA gene and a null mutation (gene deletion), is responsible for most SPTA1-related HS [1-3]. Conversely, the α LELY allele typically does not cause any significant disease, regardless of the second allele mutation, as adequate α spectrin is still being produced [10].

**Figure 2:**
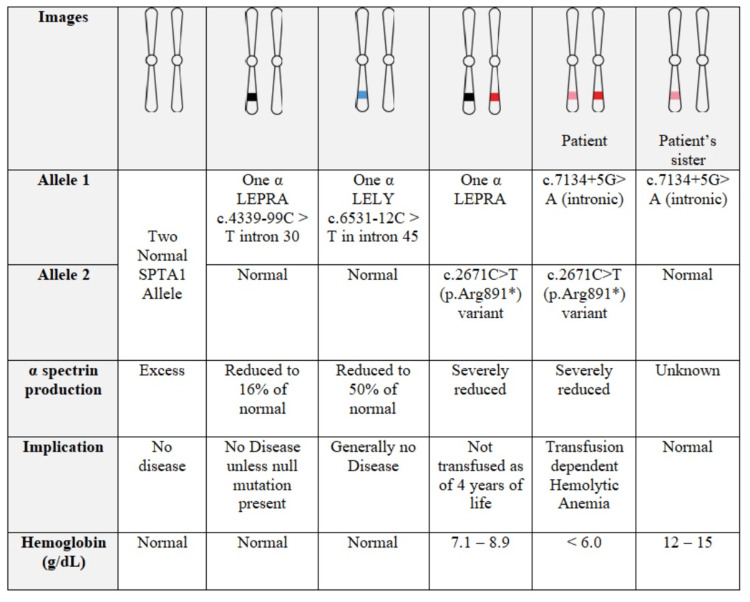
Comparison among common SPTA1-related hereditary spherocytosis and our patient.

A heterozygous mutation, involving c.2671C>T (p.Arg891*) variant and c.7134+5G>A (intronic), was found in our patient. This compound heterozygous mutation was not previously reported. This sequence change creates a premature translational stop signal (p.Arg891*) in the SPTA1 gene, resulting in an absent α-spectrin expression. SPTA1 c.2671C>T (p.Arg891*) variant was previously described in a four-year-old patient with α LEPRA mutation, who did not require any transfusion and was able to maintain her hemoglobin level between 7 and 9 g/dL [9]. Conversely, our patient was transfusion-dependent since childhood, and the presence of SPTA1 c.7134+5G>A (intronic) that was previously reported as uncertain significant may play a role in determining the severity of his disease. His sister, who had a heterozygous mutation for SPTA1 c.7134+5G>A (intronic), did not have any clinical or laboratory manifestation of the disease (Figure 2).

The transfusion-dependent status of our patient suffices as a status of severe HS. While clinical features of hemolytic anemia are readily appreciated, spherocytes are only sporadically seen in our patient’s serial blood films. The need for monthly transfusions since six months of age led to difficulty in interpreting the peripheral blood test and osmotic fragility test in diagnosing HS without a genetic study. As a result, HS was prematurely dismissed with a normal osmotic fragility test at the age of five years old without a family history of hemolytic anemia. Clinical algorithms have been described for HS, with an emphasis on next-generation sequencing (NGS), particularly in patients with clinical suspicion of hereditary hemolytic anemia to determine early diagnosis, genetic counseling, prognostication, and subsequent management [11].

As recommended, a negative osmotic fragility test should not be used solely to exclude HS, as false-negative tests can occur in up to 10% to 20% of patients [4]. The eosin-5-maleimide (EMA) binding test is now the recommended screening test. It utilizes fluorescence in flow cytometry to evaluate if RBCs are phenotypically normal by binding to specific membrane proteins, with quick results within two to three hours [4]. Osmotic gradient ektacytometry is also a good alternative, utilizing viscometer osmolality to assess the deformability of RBCs [4]. Unfortunately, neither test is routine in our public hospitals and therefore not carried out at any point during infancy and follow-up. Outsourcing to private labs was also precluded by cost and logistics concerns.

An earlier diagnosis of HS would have likely resulted in an earlier splenectomy. This would likely ameliorate the risk of secondary hemochromatosis and other complications from chronic transfusion and provide a better quality of life for our patient. Post splenectomy, the patient should be counseled for infection prevention and appropriate vaccination [3,4]. Of note, splenectomy might not be effective in patients with homozygous biallelic null SPTA mutations. These patients have absent α spectrin production, anemia, and reticulocytopenia, typically manifesting as hydrops fetalis [9,12]. They might benefit from regular transfusions, and in the presence of a suitable donor, hematopoietic stem cell transplantation (HSCT) [9].

## Conclusions

The diagnosis of hereditary hemolytic anemia has improved significantly with the availability of genetic studies. Compound heterozygous SPTA1 alleles of c.2671C>T (p.Arg891*) variant and c.7134+5G>A (intronic) have not been previously reported and potentially lead to severe disease. The role of splenectomy is well-recognized and may render the patient transfusion-free post procedure. Timely recognition and diagnosis are imperative in managing patients with HS.
